# Multiple Roles of Toll-Like Receptor 4 in Colorectal Cancer

**DOI:** 10.3389/fimmu.2014.00334

**Published:** 2014-07-15

**Authors:** Dhanusha Yesudhas, Vijayakumar Gosu, Muhammad Ayaz Anwar, Sangdun Choi

**Affiliations:** ^1^Department of Molecular Science and Technology, Ajou University, Suwon, South Korea

**Keywords:** colorectal cancer, immune response, inflammation, ligand, toll-like receptor 4

## Abstract

Toll-like receptor (TLR) signaling has been implicated in the inflammatory responses in intestinal epithelial cells (IECs). Such inflammatory signals mediate complex interactions between commensal bacteria and TLRs and are required for IEC proliferation, immune response, repair, and homeostasis. The upregulation of certain TLRs in colorectal cancer (CRC) tissues suggests that TLRs may play an essential role in the prognosis of chronic and inflammatory diseases that ultimately culminate in CRC. Here, we provide a comprehensive review of the literature on the involvement of the TLR pathway in the initiation, progression, and metastasis of CRC, as well as inherited genetic variation and epigenetic regulation. The differential expression of TLRs in epithelial cells has also been discussed. In particular, we emphasize the physiological role of TLR4 in CRC development and pathogenesis, and propose novel and promising approaches for CRC therapeutics with the aid of TLR ligands.

## Introduction

The innate immune system possesses a robust mechanism in the form of evolutionarily conserved toll-like receptors (TLRs) that can detect the signature pattern of invading microorganisms for the protection of the host. TLRs are a class of type I trans-membrane glycoproteins. Human and mouse cells comprises of 13 types of TLRs that can detect different kinds of bacterial and viral-associated patterns (1–3). TLR1–9 are highly conserved in both species; while the mouse TLR10 is non-functional due to retroviral insertion, TLR11–13 are undetected in the human genome. Examples of TLR-specific ligands are: lipopeptides for TLR1/2 and 2/6 (4–6), dsRNA for TLR3 (7), lipopolysaccharide (LPS) for TLR4 (8), flagellin for TLR5 (9), ssRNA for TLR7/8 (10, 11), and CpG DNA for TLR9 (12–14). TLRs not only detect invading microbes but also recognize intracellular anomalies and mount an immune response, thereby playing a cardinal role in the homeostasis of the human immune system (15, 16). The abnormal activation of TLRs can jeopardize normal physiological processes and cause several inflammatory diseases, cancers, and autoimmune diseases (17, 18).

Toll-like receptors are ubiquitously expressed, although their expression level may vary according to the circumstances and the tissues. In addition, induced expression of TLRs has been observed when ligands bind to their cognate TLRs (19). Research in the last decade has focused on elucidating various functions, intermediate molecules, and ligands associated with TLRs. There is a well-established link between TLR-induced inflammation and the development and progression of cancer (20, 21). Similarly, TLRs are also known to play a vital role in colorectal cancer (CRC) that affects the large intestine and the rectum. This region is heavily populated by intestinal microbes, highlighting the crucial role of TLRs in CRC pathogenesis (17, 22).

Colorectal cancer is one of the most complex diseases and causes death in many cases in the United States (23). Globally, more than one million new cases of CRC are reported annually (24, 25). The complexity of CRC is primarily attributed to environmental factors, while genetic factors play a minor role. The known risk factors for CRC are food-borne mutagens, pollution, certain commensal bacteria, and chronic intestinal inflammation (25). Commonly, CRC occurs in the right ascending colon with the most common symptom being blood in the stool or rectal bleeding. Genetically, inherited colon polyps also contribute to the development of CRC (26). Since CRC can damage the host immune system during their proliferation period, stimulating it against CRC promises to be an attractive approach for drug discovery (27).

In this review, we discuss the role of TLRs in the maintenance of homeostasis and the development of CRC in intestinal epithelial cells (IECs). Improved techniques to detect dysfunctional TLR signaling in carcinogenesis may stimulate the development of novel therapies to prevent or treat CRC. Recent studies have improved the understanding of TLR-targeted applications such as identifying their differential expression, their role in tumor progression, potential use as immune modulating agents, and development of novel TLR ligands in anti-cancer therapies.

## TLR Signaling: An Overview

**Figure 1:**
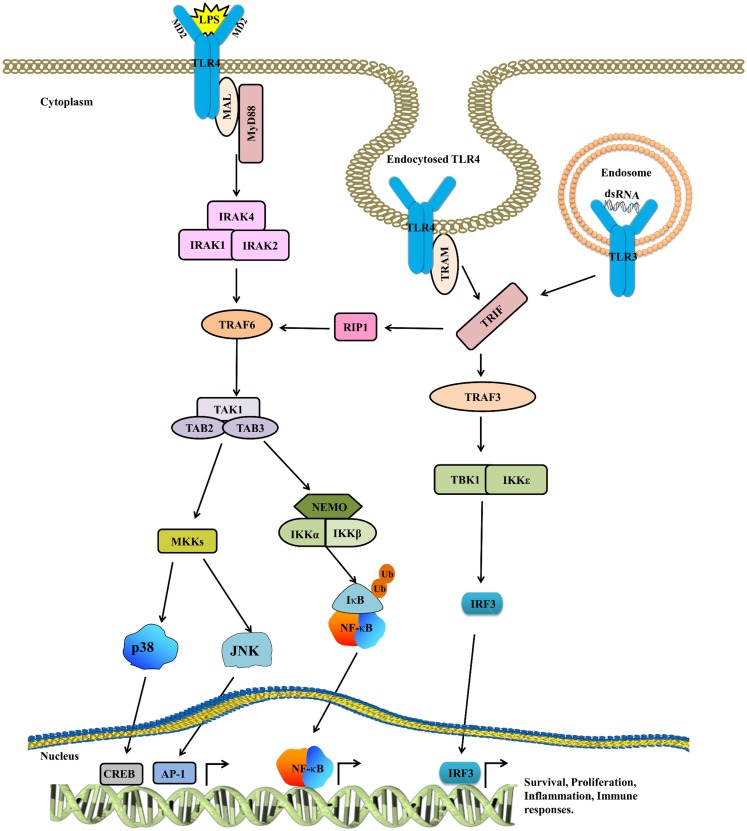
**The TLR4 signaling pathway**. TLR4 is activated by LPS, whereas CD14 and MD2 act as accessory proteins for LPS/TLR4 binding. Upon ligand binding, TLR4 dimerizes, and recruits downstream adaptor molecules such as MyD88/MAL and TRIF/TRAM to mount an inflammatory response. The activated MyD88/MAL then activates IRAK4, TRAF6, TAK1, and IKK complexes, while TRIF/TRAM signals through RIP1 to TRAF6/TAK1 and IKK. After this, both these pathways converge at NF-κB. The cytoplasmic NF-κB complex is maintained in the inactive state by IκB, which is in turn degraded by proteasomes, resulting in the translocation of NF-κB into the nucleus. Besides activating NF-κB, TAK1 also phosphorylates MAPKs to further reinforce the inflammatory response. The TRIF/TRAM pathway not only activates NF-κB but also triggers IRF3 to mount an antiviral response. Cumulatively, all these signaling pathways assist in eradicating infection as well as play an important role in sustaining the normal physiological functions in IECs.

The localization of TLRs is heterogeneous and varies from the cell surface (TLR1, 2, 4, 5, 6, 10, and mouse TLR11, 12) to the endosomes (TLR3, 7, 8, and 9) (28), depending on the localization of pathogen-associated molecular patterns (PAMPs). TLRs comprises of the following three domains: ectodomain [contains leucine rich repeats (LRR)] that recognizes PAMPs, a trans-membrane region, and a cytosolic toll/interleukin-1 (IL-1) receptor (TIR) domain that interacts with adaptor molecules (such as MyD88/MAL and TRIF/TRAM) to propagate downstream signaling. Ligand binding triggers the dimerization of TLRs, facilitating the binding of adaptor molecules, which subsequently activate the IL-1 receptor-associated kinase (IRAK) family (29). Upon IRAK recruitment, IRAK4 phosphorylates IRAK1 at key serine and threonine residues, and enables IRAK1 to eventually activate tumor necrosis factor receptor-associated factor 6 (TRAF6) (30) that subsequently activates transforming growth factor-β-activated protein kinase 1 (TAK1), a member of the mitogen-activated protein (MAP) kinase kinase kinase (MAP3K) family. TAK1 forms a complex with TGF-β-activated kinase 1/MAP3K7 binding protein 1 (TAB1), TAB2, and TAB3 and then activates nuclear factor (NF)-κB by phosphorylating IKK that in turn phosphorylates IκB for proteasomal degradation. Following the degradation of IκB, NF-κB translocates into the nucleus and induces inflammatory mediators. Moreover, TAK1 activates members of the MAP kinase kinase 3 (MKK3) and MKK6 to activate an alternative closely related pathway that phosphorylates c-Jun N-terminal Kinase (JNK) and p38. TLR signaling can also activate extracellular signal-regulated kinase (ERK) via the activation of MEK1/2. In response to various TLR ligands, reduced activity of NF-κB, JNK, and p38 was observed in B cells and embryonic fibroblasts derived from TAK1-deficient mice (31). In the TRIF-dependent pathway triggered by TLR3 and TLR4, TRIF recruits TRAF3, TAB1, and IKK and activates the type I IFN. The TRIF-dependent pathway also activates TRAF6 and TAB1, which regulate the delayed activation of NF-κB and MAP kinases (32) (Figure 1).

## TLRs and Their Expression Patterns in IECs

The human intestinal tract plays a crucial role in maintaining the complex ecosystem of commensal bacteria and also physically isolates the countless resident bacteria from the lamina propria (33). It was originally believed that IECs prevent bacteria from invading the body. However, IECs have a complicated and common beneficial link with the microorganisms in the intestinal gut flora. The commensal bacteria metabolize carbohydrates and the IECs break down the short-chain fatty acids produced as a result of bacterial fermentation of undigested carbohydrates and use them as an energy source (34). IEC membranes express TLRs that detect the commensal PAMPs and mediate signaling to maintain epithelial cell integrity and tight junctions, cell proliferation, immunoglobulin A (IgA) production, and antimicrobial peptide expression (34). In addition, they can also induce a pro-inflammatory response by interacting with the immune cells in the lamina propria (35, 36). Therefore, tight regulation of TLRs is imperative to prevent adverse effects since anomalous or dysregulated TLR signaling can mediate cancer induction and propagation.

Colorectal cancer pathogenesis is governed by TLR expression that is difficult to detect due to the heterogeneous nature of IECs (33). To elucidate the expression profile of TLR2–5 in epithelial cells, small intestinal, and colonic biopsy specimens from patients with inflammatory bowel disease (IBD) were assessed by immunofluorescence histochemistry using polyclonal antibodies against TLR2, 3, 4, and 5. This study showed that TLR3 and TLR5 are ubiquitously expressed while TLR2 and TLR4 are expressed at a very low level in normal cells (37). Conversely, the diseased tissue specimens demonstrated significant overexpression of TLR4 and a decline in TLR3 expression. The expression pattern of TLR2 and TLR5 remained unaltered between the normal and diseased specimens. Furthermore, in normal human IECs, TLR2, and TLR4 were marginally expressed, while TLR3 expression was relatively high. While TLR2 was expressed in the colonic tissue from the epithelium and lamina propria, TLR3 was expressed in the mature epithelial cells of the crypts. Furthermore, TLR5 was moderately overexpressed in a basolateral fashion in the epithelial cells of normal human tissues (38). Tissues from CRC patients demonstrated increased expression of TLR7, 8, 9, and 10 (39); this study also showed that TLR8 expression is an independent marker for CRC.

## TLRs and Intestinal Homeostasis

Toll-like receptor activation is responsible for fighting against microbial infections, while leaving the host cell intact. This is usually accomplished by producing antimicrobial peptides, inflammatory mediators, adenomatous polyposis coli (APCs) maturation, and triggering of cell survival and tissue repairing pathways (40). TLRs are marginally expressed on IECs and are primarily localized on the basolateral surface or in the endosomal vesicles (41). Moreover, regulatory mechanisms such as the expression of TLR inhibitors like single immunoglobulin IL-1-related receptor (SIGIRR), toll-interacting protein (TOLLIP), A20, and IRAK3 are involved in the regulation of TLR signaling (42); these inhibitory molecules prevent TLRs from mounting an immune response even during continuous interaction (34, 43) and nurturing the anti-inflammatory phenotype of homing leukocytes (44). SIGIRR-deficient mice demonstrate defective intestinal homeostasis, and these defects are associated with the microbiota and hyper-expression of inflammatory mediators. Notably, these defects also render the azoxymethane (AOM)-dextran sodium sulfate (DSS)-treated SIGIRR^−/−^ mice prone to colitis and colitis-associated CRC. Interestingly, the rescue of SIGIRR expression in the IECs of SIGIRR^−/−^ mice restored the immune tolerance and abolished the risk of tumor development in these mice (45).

Although elevated TLR activity disrupts the recognition of intestinal microbes by TLR2 and TLR4, TLR signaling is necessary for maintaining homeostasis and regulation of tissue repair in IECs. MyD88-deficient mice, which hamper signaling through IL-1 family members including TLRs, possess profound abnormalities in the mucosa with higher proliferation rates in the crypts (46). Cumulatively, this leads to defects in repair of the intestinal barrier following injury, and increased risk of colitis and CRC (46, 47). Moreover, mice in which normal flora is disrupted by antibiotics display a similar phenotype to mice lacking MyD88, as well as decreased expression of factors [i.e., tumor necrosis factor (TNF), CXC-chemokine ligand 1, IL-6, and heat shock proteins] required for normal intestinal homeostasis (48).

## Relationship between Inflammation and CRC

The direct link between intestinal inflammation and CRC prognosis is well-established and is also supported by numerous genetic, pharmacological, and epidemiological studies conducted during the last decade (49). Recent reports demonstrate the complex interplay between distinct immune cells, and also show that pro-inflammatory mediators influence almost all the steps of CRC progression. However, the mechanisms by which inflammation stimulates the development of cancer remain elusive and are expected to vary from colitis-associated CRC to other forms of CRC (25, 50). The relationship between inflammatory responses caused by multiple factors such as the microbiota, IBD, and CRC has been demonstrated by comparative experiments conducted in wild type and *Il10*^−/−^ mice. When treated with AOM, *Il10^-/-^* mice were found to show an increased risk of colon tumor development, spontaneous colitis, and CRC, while AOM-WT mice were devoid of colitis and rarely progressed to adenomas. In addition, mice with *Bacteroides vulgatus* or dual knockout mice (*Il10-* and *MyD88-*deficient mice) treated with AOM showed reduced transcription of *Il12p40* and *TNF-*α and remained tumor-free (51).

TLR-induced inflammation is a well-established phenomenon and is perpetuated by several cytokines, ILs, and TNF-α, all of which are known to substantially regulate immune cells and inflammatory responses against cancer (48, 52). Among these, TNF-α is of particular importance and is now recognized as a pro- as well as anti-tumorigenic protein (53). The activation of the TLR4 signaling pathway induces TNF-α and NF-κB, leading to the promotion of CRC (17, 54–56); TNF-α knockout mice treated with AOM/DSS show significantly less tumor formation, representing the pro-tumorigenic role of TNF-α (57). Immunohistochemistry analyses of mononuclear cells in the lamina propria and colons of patients with advanced stage CRC demonstrate the expression of TNF-α (57). TNF-α also promotes the activation of NF-κB, which reinforces inflammation by inducing cyclooxygenase-2 (COX-2), IL-6, IL-8, and TNF-α to favor tumorigenesis (55, 58, 59). However, inflammation alone is not sufficient for colon cancer and the contribution of other risk factors is equally essential to the pathogenesis of this complex disease.

## Contribution of TLR4 to CRC Development

**Figure 2:**
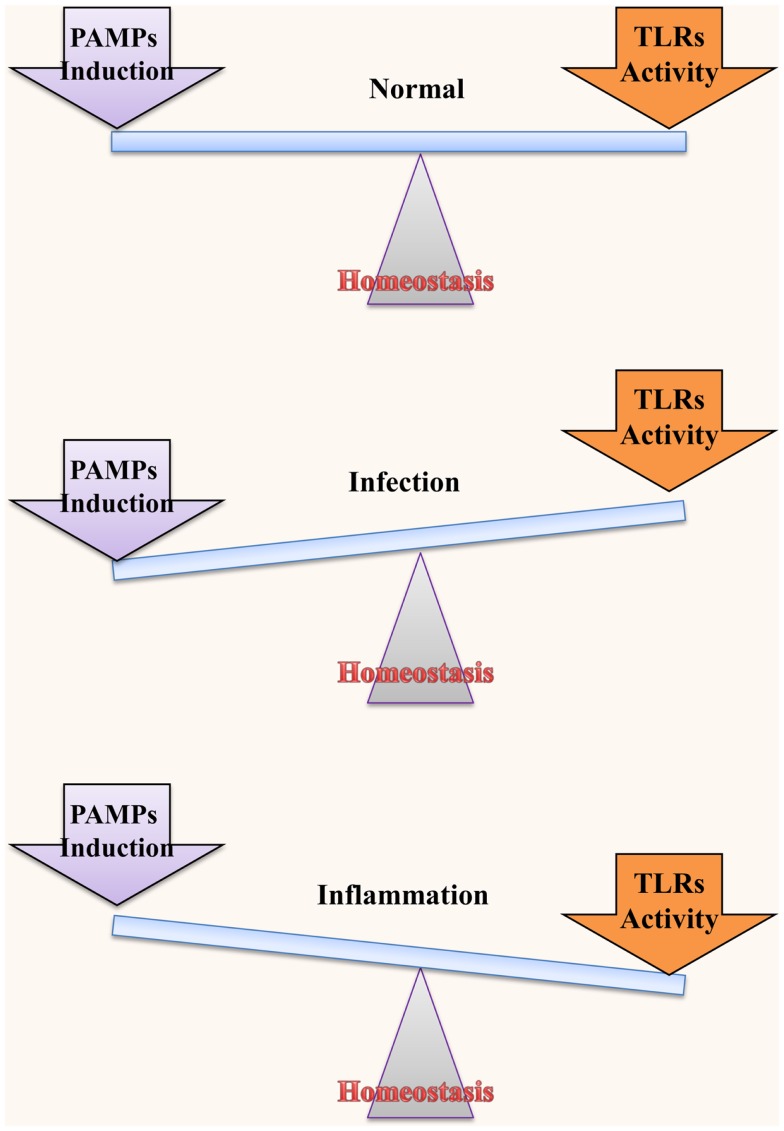
**Homeostatic interaction between microbiota and TLRs**. TLRs play an important role in maintaining normal functions of IECs; however, regulation of the activation and induction of TLRs through various mechanisms is necessary for this role. TLRs in the intestine exist in close proximity to and may be stimulated by commensal bacteria. Therefore, it is extremely necessary to regulate their functions. Under normal conditions, homeostasis between bacterial induction and TLR activation is maintained to ensure a disease-free status. On the other hand, if TLRs are inappropriately activated or if they mount an exaggerated immune response to a low level stimulus, they may culminate in bacterial infection and inflammatory disease/cancer, respectively.

Although IECs are in close proximity to LPS, they do not mount an immune response on the commensal bacteria under normal circumstances. However, in the diseased state, disruption of the coexistence between IECs, and bacteria leads to an inflammatory response. This raises an important question: when and how much inflammation should have to be raised in order to equilibrate the bacterial threat (Figure 2). Numerous studies have been conducted to address this dilemma (60–64). For instance, IFN-α and IFN-γ are known to increase the LPS response in IECs, which is directly linked to the expression of TLR4 and MD2 (63, 65). Moreover, continuous LPS stimulation culminates in reduced TLR4 expression and increased expression of inhibitory proteins (62). However, a conflicting report demonstrated that long-term LPS exposure does not alter TLR4 expression (66). Moreover, hypoxia and numerous endotoxins are known to be prevalent in the inflamed intestinal lining, possibly causing induced TLR4 expression (60). Hung et al. observed an increase in the TLR4 expression from the mucosa of CRC patients of different ages and sexes as well as from a variety of CRC cell lines (HT29, SW480, and KM20) (67). In addition, Maria and colleagues showed that TLR4 expression is required for dysplasia and polyp formation. This finding is consistent with results of experiments performed in TLR4 gene knockout mice (56, 68). Collectively, these data present a clear association between TLR4 and CRC development.

In CRC, elevated TLR4 expression is observed in all tumor components such as the epithelial, endothelial, and stromal layers (69). However, the level of this expression varies depending on the type of cancer. Although all TLRs are expressed at the minimal basal mRNA level, IECs can upregulate the TLR expression, based on the inflammatory signals or other stimuli (70). An alternate study demonstrated a low level of TLR4/MD2 expression in normal human colonic epithelial cells and the lamina propria, which is consistent with the level of TLR4/MD2 expression detected in various epithelial cell lines (71). These studies establish the fact that any alteration in the prevalent inflammatory conditions or the population of luminal bacteria may influence the strength and nature of TLR signaling, paving the way for initiation of inflammatory responses in IECs. Besides this negative role, studies in TLR4- and TLR9-deficient mice have shown that TLR signaling in IECs is essential for protecting the host from inflammation-related damage and for homeostasis (46, 72).

## TLR4 Crosstalk in CRC Progression

TLR4 is overexpressed in the liver metastasis of CRC (73). In response to LPS binding, over-stimulation of the TLR4/MD2 complex enhances the phosphorylation of protein kinase B (also known as AKT), which in turn activates the function of β1 integrin. This complex interplay between multiple pathways promotes the adhesiveness and metastatic behavior of CRC (74). The enhanced AKT phosphorylation can be blocked by eritoran (a TLR4 antagonist), PI 103 [a phosphatidylinositide 3-kinases (PI3K) inhibitor], or anti-β1 integrin antibodies that are known to ameliorate CRC and its metastatic behavior (75–77), indicating that the PI3K/AKT signaling pathway is induced by TLR4 in response to LPS binding and plays a central role in the growth and progression of CRC. Furthermore, LPS is known to induce the expression of the urokinase plasminogen activator (uPA) system through TLR4 and NF-κB in human colorectal cell lines. During tumor progression, vital extracellular matrix (ECM) interactions occur, in which uPA and the expression and activity of its receptor facilitate the growth and metastasis of CRC (78). Conversely, inhibition of TLR4, NF-κB, or the uPA system can attenuate CRC progression. Although NF-κB is known to impair apoptosis in tumor cells (55, 79, 80), NF-κB activation through TNFR signaling also protects cells from apoptosis. Studies performed in the Saos-2 cell line reveal that p53-induced cell death is dependent on NF-κB, and the ablation of NF-κB leads to the abrogation of p53-dependent cell death (81). Thus, the TNF-α/NF-κB interaction plays a vital role in CRC and IBD-related diseases and manipulation of this interaction may improve the treatment of CRC.

TLR4 is overexpressed during inflammation-associated colorectal neoplasia in humans and mice. Similarly, mice lacking TLR4 are largely protected from colon carcinogenesis (56). A dissection of this mechanism reveals that TLR4 triggers elevated production of prostaglandin E2, increases Cox-2 induction, and influences epidermal growth factor receptor signaling (EGFR) in chronic colitis. TLR4 can thus manipulate numerous pathways and cause further deterioration of the neoplastic situation. A recent comparative immunohistochemistry analysis between normal mucosa and adenomas showed that TLR4 and MD2 are overexpressed in 20 and 23% of the adenomas, respectively (82), further substantiating the involvement of the TLR4 pathway in CRC. Furthermore, mutations in the *APC* gene cause pre-disposition to CRC. A correlation between the TLR/MyD88 signaling pathway and *APC* mutations was recently proposed (82, 83) since MyD88 signaling was found to facilitate the growth of intestinal polyps while the ablation of MyD88 restricted polyp growth in *Apc*^min/+^/*Myd88*^−/−^ mice, but not in *Apc*^min/+^ mice (83, 84). In addition, MyD88 induces ERK to block the degradation of the oncoprotein c-Myc, and such cells with continued activation of c-Myc are prone to neoplastic transformation (85). Similarly, c-Myc is also important for APC-mediated tumorigenesis (86), since knocking out c-Myc in IECs of *Apc*^min/+^ mice impedes tumor growth (84). Furthermore, reduced expression of c-Myc has been reported in *Apc*^min/+/^*Myd88*^-/-^ of both normal and tumor mice (84, 87). Treatment of *Apc*^min/+^ mice with PD03259012, an inhibitor of MEK1/2, which is the kinase directly upstream of ERK, also inhibits tumor growth. These data indicate that a complex interplay of protein signaling brings about tumor proliferation in the IECs of various transgenic mouse models. Moreover, heritable changes in the *APC* gene frequently lead to familial adenomatous polyposis (FAP). FAP is the most dominant inherited syndrome of CRC (88, 89) and *Apc*^min/+^ mice show increased propensity for the development of adenomatous polyps after the loss of the wild type *APC* allele (88). Up to 80% of sporadic CRCs are known to be initiated by DNA damage of the genes involved in the APC signaling pathway (87).

## Correlation between CRC Development and Inherited Genetic Variations of TLR4

The human *TLR4* gene is located on the long (q) arm of chromosome 9 at position 33.1, and contain four exons. The dominant expression of TLR4 has been observed in lymphocytes, monocytes, leukocytes, and splenocytes (90). Besides CRC, many human pathologies and carcinomas are associated with the polymorphisms of TLR4 (91–93). The *TLR4* gene contains two single-nucleotide polymorphisms (SNPs), namely, Asp299Gly and Thr399Ile that are significantly important in tumor development (94, 95). Both these SNPs are located in the coding sequence for the TLR4 ectodomain and mediate an amino acid substitution. These Asp299Gly and Thr399Ile SNPs in *TLR4* are known to attenuate cytokine expression, leading to an increased propensity for the development of gastric cancer and CRC (94, 96–99). The detection of these two SNPs was carried out using allele-specific polymerase chain reaction and the primer extension method (SNaPshot) for gastric cancer and CRC, respectively. For gastric cancer, only Thr399Ile showed a significant correlation, while both the SNPs were significantly correlated to CRC (94, 100, 101). In addition, the association of the TLR3 (rs3775291) polymorphism and IL-10 promoter variation (rs1800872) to CRC pathogenesis was evaluated in a large cohort of German CRC patients. This study found that the IL-10 promoter variant is significantly associated with an increased risk of lymph node metastasis (for carriers of the TT genotype). Interestingly, a *TLR3* gene polymorphism was found to correlate with patient survival, and the TT genotype was responsible for increased mortality. This TLR3 variation was limited only to stage II patients who were devoid of adjuvant therapy (102, 103).

The LPS-sensing complex is comprises TLR4, MD2, LPS binding protein, and CD14. A positive link between CD14–260 polymorphisms and the occurrence of CRC in the Chinese Han population was demonstrated (104, 105), in which the CD14 polymorphism C/C, but not C/T, was significantly correlated to CRC; no correlation between TLR4 Asp299Gly and CRC was found. However, it is possible that the polymorphism in TLR4 was associated with the population under study (106). A multi-racial study (22 Malays, 20 Chinese, and 18 Indians) conducted in Malaysia showed that there is no correlation between TLR4 polymorphisms (Asp299Gly; Thr399Ile) and the risk of CRC (107). However, a study on Russian population revealed that IL1B_1473G/C and TLR4_896A/G SNPs are involved in rectal cancer development (108). A conflicting report validated the potential link between TLR4 polymorphisms (Asp299Gly and Thr399Ile) and the digestive tract cancer and CRC (101). This study retrieved and analyzed extensive data from various databases and concluded that Asp299Gly is significantly correlated with an increased risk of gastric cancer, while there was no correlation between this polymorphism and digestive tract cancer and CRC. Moreover, it was also observed that the T allele of Thr399Ile does not influence digestive tract, gastric, or CRC. It is evident that additional studies are necessary to support these findings.

## Epigenetic Regulation of TLR4 in CRC

Intestinal epithelial cells are stimulated by the commensal bacteria in the intestinal lumen with the help of TLRs for the maintenance of homeostasis. This stimulation from the commensal bacteria is finite, should not trigger an excessive inflammatory response, and is known to influence epigenetic modification in the host cells (109). These epigenetic modifications involve DNA methylation and histone deacetylation that suppress and promote the transcription process, respectively, and in turn regulate gene expression (110).

The *TLR4* gene is methylated in the 5′ region; also, the degree of methylation in epithelial cells is higher than that in the splenic cells, caused by the interaction of the commensal bacterial with IECs. Takahashi and colleagues showed that commensal bacteria modulate the epigenetic regulation in IECs by DNA methylation of *TLR4* (111). In their study, the authors compared the methylation levels in the IECs from the small and large intestine obtained from conventional (CV) mice with commensal bacteria and germ-free (GF) mice without commensal bacteria. The methylation level of CpG motifs in the 5′ region of *TLR4* from the large intestine was lower in the GF mice compared with CV mice, while in the small intestine, the methylation levels remained unchanged between the GF and CV mice. The frequency of methylation is also found to depend on the MyD88 adaptor molecule. Results from *in vivo* experiments show that the frequency of CpG methylation is less in the GF mice (MyD88 knockout mice) compared to CV mice (111).

Environmental factors also play a crucial role in regulating epigenetic modifications. In the presence of factors such as myriad food habits and increasing pollution, intestinal commensal bacteria produce short-chain fatty acids known as butyrates that inhibit histone deacetylation (112, 113). Besides *TLR4, MD2* can also be downregulated to attenuate the LPS response. IECs are known to poorly express *MD2*, which directly correlates to DNA hypermethylation (114). In IBD, IECs exhibit elevated expression of *MD2* and *TLR4* mRNA, while in normal cells; *TLR4/MD2* transcription is reduced due to DNA methylation. The deacetylation and blocking of methylation enables cells to express higher amounts of *TLR4* and *MD2* mRNA. This study demonstrates how epigenetic regulation of *TLR4* and *MD2* prevents dysregulation of inflammation in IECs and thus provides a novel approach to target CRC.

## Therapeutic Targeting of TLR4

Synthetic TLR4 ligands are potential targets for therapeutic applications for cancer, allergies, and viral infections (115). By virtue of their cell surface location, quick induction, and the ability to mount a wide array of inflammatory responses, TLRs are one of the most promising targets for therapeutics (91). The clinical trials of various TLR4 ligands are enlisted in Table 1.

**Table 1:** **TLR4 agonists in clinical trials**.

Compounds	Phase	Note	Indications	Current status	Clinical Trail.gov
LPS	I–II	Combined with KLH-pulsed DCs vaccine	Neuroblastoma and Ewing’s sarcoma	Active, not recruiting	NCT00923351
	I–II	Combined with IL-4, KLH, and WT1 peptide-pulsed DC based vaccine	Hematologic malignancies	Completed	NCT00923910
	I	Combined with multipeptide vaccine	Melanoma	Active, not recruiting	NCT01585350
OM-174	I	Injections of OM-174	Solid tumors	Completed	NCT01800812
Stimuvax	II	Combined with chemoradiation therapy	Rectal cancer	Active, not recruiting	NCT01507103
	II	Androgen deprivation and radiation therapy	Prostate cancer	Recruiting	NCT01496131
	II	L-BLP25 vaccination	Colorectal carcinoma	Recruiting	NCT01462513
Picibanil	IV	Intracystic injection	Cystic malformation	Recruiting	NCT01699347
	I–II	Combined with pre-operative intra tumoral DCs	Pancreatic cancer	Unknown	NCT00795977
	I	Combined with cyclophosphamide, docetaxel (chemo-immunotherapy)	Head and neck cancer	Unknown	NCT01149902

TLR4 agonists have immune regulatory applications as adjuvants in vaccines and in the treatment of chronic viral infection and cancer therapy. LPS was the first microbial product identified as a potential TLR4 agonist and implemented for therapeutic applications (116). LPS is very toxic since it induces excessive inflammatory cytokines. However, low-dose LPS combined with non-steroidal anti-inflammatory drug ibuprofene was proved to be safe, with higher levels of TNF-α and IL-1 in all patients (117, 118). Marginal to encouraging results were observed when ibuprofene combined with *Salmonella abortus equi* LPS for non-small cell lung carcinoma (NSCLC) and CRC patients, respectively (119). Currently, a few clinical trials are being conducted for oncological indications involving cell-based vaccination to treat Ewing sarcoma, neuroblastoma, and rhabdomyosarcoma patients (NCT00923351). Besides, peptide-pulsed dendritic cells (DCs) were combined with LPS to treat hematological malignancies (NCT00923910), and to treat melanoma patients (NCT01585350), LPS along with oil-based adjuvant and a peptide vaccine are being investigated (119). A less toxic TLR4 agonist, monophosphoryl lipid A (MPLA), is an immunity modulating agent that activates MyD88-independent pathway in TLR4 signaling, triggers the induction of IFN-γ, and regulation of CD80/86, which forms the crucial aspect of adjuvancy (27, 120). MPLA adjuvant plays a dual role in defending the host from pathogens by stimulating the innate immune system, and induces the long-term adaptive immune system (115, 121, 122). Food and Drug Administration has approved MPLA to use as a vaccine against HPV associated cervical cancer (Cervarix). It also enhances the inflammatory behavior of immune cells, which may be useful in a variety of cancers to overcome the cancer-induced immune suppression. However, this may not be helpful in case of CRC, where TLR should emphasize the tolerance of immune system, not the over-activation. Furthermore, it is established that TLRs can act as double-edge sword that may be exploited in pathologies-dependent circumstances to avoid the undesirable consequences (123). OM-174 is a triacyl lipid A analog that activates TLR4 and culminates in tumor growth regression by increasing the IFN-γ production (124, 125) This is well-tolerated at biological concentrations with strong antitumor effects (NCT01800812) (126). A new anti-cancer vaccine, BLP25 liposome vaccine (Stimuvax), can identify and destroy the cancer antigen MUC1, thereby inducing an immune response against cancer cells (127, 128). However, this could not significantly improve the NSCLC (128). Now, stimuvax is being investigated for the treatment of rectal and prostate cancers (NCT01507103 and NCT01496131). Group A *Streptococcus pyogenes* [in lyophilized form OK-432 (Picibanil)] is shown to stimulate TLR4, which is used to treat gastric, cervical, and oral cancers (119). This compound is currently being examined to treat pancreatic cancer patients in pre-operative settings using intra tumoral injection of DCs (NCT00795977), combined with chemotherapy (cyclophosphamide + docetaxel) for head and neck squamous cell carcinoma (HNSCC) patients (NCT01149902), and via intracystic injection at cystic malformation (NCT01699347). Currently, most of the TLR4 antagonists are being evaluated against cancer-unrelated symptoms.

## Conclusion and Future Perspectives

In this review, we highlight the correlation between CRC and TLRs, in particular, TLR4. We also propose that a beneficial link exists between commensal bacteria and TLRs in order to maintain intestinal homeostasis. In IECs, TLRs are involved in epithelial cell proliferation, IgA production, regulating the permeability of the intestinal barrier, antimicrobial peptide expression, and defense against invading pathogens. Over-stimulation of TLRs in response to minor signals (due to dysregulation) may result in colitis and CRC. Several studies suggested the relationship of TLR4 signaling with CRC, therefore therapeutic benefit can be achieved by targeting TLR4. However, the development of CRC is highly complex. Experimental studies supported that the gut microbiota contributed to CRC. The studies involving human subjects and considering their microbiota composition revealed the vivid differences in microbial density and population. Therefore, modulating the microbial population, usage of probiotics to favor the growth of certain bacteria, and delineating the interaction of microbiota with the epithelial cells can potentially be used to limit the CRC development.

Furthermore, inflammation is central to the development of cancer, and there are few clinical trials being conducted for anti-inflammatory drugs, but by combining molecular approaches with CV therapies, i.e., chemo- or radiotherapy, anti-inflammatory drugs would increase the efficacy to treat CRC. Additionally, targeting the downstream molecules in TLR4 pathway involved in CRC is also expected to have a tremendous impact on CRC therapeutics. Moreover, differential expression of TLRs leads to tumor development, in which the contribution of TLR4 is considerably higher than in the other TLRs. We hope that extensive studies involving the TLR4 pathway will eventually provide therapeutic targets to treat CRC. Recently developed techniques may also prove helpful in the analyses of differential expression levels of TLRs, their mutations, and epigenetic modifications. These analyses would further aid in the design and development of novel therapeutic approaches for CRC treatment.

## Conflict of Interest Statement

The authors declare that the research was conducted in the absence of any commercial or financial relationships that could be construed as a potential conflict of interest.
